# Antioxidant potential of the methanol–methylene chloride extract of *Terminalia glaucescens* leaves on mice liver in streptozotocin-induced stress

**DOI:** 10.4103/0253-7613.45153

**Published:** 2008

**Authors:** Guy Bertrand Sabas Nya Njomen, René Kamgang, Jean Louis Essame Oyono, Njifutie Njikam

**Affiliations:** General Endocrinology and Metabolism Systems (GEMS), Laboratory of Animal Physiology, Faculty of Sciences, University of Yaounde 1, Cameroon; 1 Faculty of Medicine and Biomedical Sciences, University of Yaounde 1 and IMPM – Yaounde, Cameroon

**Keywords:** Antioxidant enzymes, diabetes mellitus, malondialdehyde, *Terminalia glaucescens*

## Abstract

**Aim:**

The antioxidant effect of the methanol–methylene chloride extract of *Terminalia glaucescens* (Combretaceae) leaves was investigated in streptozotocin (STZ)-induced oxidative stress.

**Methods:**

Oxidative stress was induced in mice by a daily dose of STZ (45 mg/kg body weight i.p.) for five days. From day one, before STZ injection, normal and diabetic-test mice received an oral dose of the extract (100 or 300 mg/kg b.w.) daily. Plasma metabolites, lipid peroxidation, and antioxidant enzymes in the liver were assessed and gain in body weight recorded.

**Results:**

In normal mice the plant extract reduced food and water intake, blood glucose and LDL-C level and body weight gain, did not affect the lipid peroxidation in the liver, while the antioxidant enzyme activities seemed increased. Blood glucose was decreased (*P* < 0.05) in normal mice treated with 300 mg/kg extract. Diabetic mice pretreated with 100 mg/kg extract as diabetic control mice (DC) showed significant (*P* < 0.001) body weight loss, polyphagia and polydipsia, high plasma glucose level, decrease in the liver catalase, peroxidase, and superoxide dismutase activities, and increase in lipid peroxidation. The HDL-C level was lowered (*P* < 0.05) whereas LDL-C increased. In 300 mg/kg extract-pretreated diabetic mice the extract prevented body weight loss, increase of blood glucose level, lipid peroxidation in liver, food and water intake, and lowering of plasma HDL-C level and liver antioxidants; this extract prevented LDL-C level increase.

**Conclusion:**

These results indicate that *T. glaucescens* protects against STZ-induced oxidative stress and could thus explain its traditional use for diabetes and obesity treatment or management.

## Introduction

Diabetes mellitus is a major endocrine disorder and growing health problem in most countries.[1] Diabetes manifested by experimental animal models exhibit high oxidative stress due to persistent and chronic hyperglycemia which increases the generation of free radicals, thereby depleting the activities of antioxidative defense systems with alteration of antioxidant activities of enzymes such as superoxide dismutase (SOD), catalase (CAT), and glutathione peroxidase (Gpx).[2] Increase in oxidative stress and changes in antioxidant capacity are the main participants in the development of diabetic complications.[3] In diabetes there are significant changes and irregularities in the metabolism of proteins, lipids, and carbohydrates. Streptozotocin (STZ) is frequently used to induce diabetes mellitus in experimental animals through its toxic effects on pancreatic β-cells.[4,5] The cytotoxic action of STZ is associated with the generation of reactive oxygen species causing oxidative damage.[6]

Some plants or natural substances protect against the death of β-cells that precedes diabetes.[7] *Terminalia arjuna*, *T. catappa,* and *T. chebula* (Combretaceae) are known for their antioxidant properties.[8,9] In Cameroon, *T. glaucescens* is claimed to be useful in the treatment of diabetes mellitus, obesity management, and some bacterial diseases. The preliminary phytochemical screening of this plant extract revealed the presence of tannins, alkaloids, flavonoids, and saponosides.[10] Among the natural antioxidant substances, pride of place was given to flavonoids, ubiquitously present in the plant kingdom and which exert antioxidant, anti-inflammatory, and lipid lowering effects.[11]

Although many plants offer certain medical benefits to humans, many of these claims are unproven scientifically. The objective of the present study was to assess *in vivo* the protective role of *T. glaucescens* extract on the liver of the mice in multiple low dose STZ-induced oxidative stress.

## Materials and Methods

### Plant material and extract

*T. glaucescens* (Combretaceae) fresh leaves were harvested from Mbalmayo in the Centre Province of Cameroon. *T. glaucescens* was identified by Dr. Simeon Tchoulagueu of the Teachers’ Training Higher College of the University of Yaoundé I, who did the botanical study of the plant and kept a voucher specimen in the laboratory. Two kilograms of the sun-dried powdered leaves were macerated in a mixture of methanol–methylene chloride (1:1) for seven days (with occasional stirring) at room temperature. The mixture was filtered with Whatman No.1 filter paper. The filtrate was concentrated under reduced pressure to obtain 125 g of a dark solid. This extract was dissolved in 10% dimethyl sulfoxide (DMSO) solution. The volume of administration was 5 μL/g b.w. for each experimental animal.

### Animals used for the experiment

Male albino Wistar mice (26–30 g weight, 8–10 weeks old) were raised in the animal house of the laboratory under natural conditions with free access to water and regular rodent chow. For the experiment, the mice were fasted overnight prior to blood sugar determination and randomly divided into six groups of eight animals each:

- One group of normal control (NC) receiving 10% DMSO (p.o.) and citrate buffer (Cb), i.p.
- Two groups of normal mice treated with 100 mg/kg body weight (NE100) or 300 mg/kg b.w. (NE300) plant extract (p.o.) and Cb (i.p.).
- One group of diabetic control mice (DC) treated with DMSO and STZ.
- Two groups of diabetic mice treated with 100 mg/kg b.w. (DE100) or 300 mg/kg b.w. (DE300) plant extract and STZ.

To induce diabetes, the mice received 45 mg/kg (i.p.) of freshly prepared STZ (Sigma Aldrich No. SO 130) dissolved in Cb of 100 mM pH 4.5 daily for five consecutive days. Plant extract was administered to fed mice when STZ injection was preceded by 4 h of fasting. *T. glaucescens* extract administration began one day prior to STZ injection (day 0) and lasted up to day 14. Animals DE100 and DE300 received an oral dose of plant extract daily for five consecutive days followed six hours later by i.p. injection of STZ.

Animal housing and *in vivo* experiments were done according to the guidelines of the European Union on Animal Care (CEE Council 86/609) that was adopted by the Institutional Committee of the Ministry of Scientific Research and Innovation of Cameroon.

### Measurement of body weight gain, food, and water intake

Food and water intake were monitored on day 0, 1, 3, 6, and 12. Body weight was measured on day 0, 3, 6, 9, 12, and 15.

### Determination of plasma metabolites

Blood samples for glucose determination were obtained from the tail tip of 4-h fasted mice on day 0, 3, 6, 9, and 12 of the experiments. Blood glucose level was estimated using a glucometer (Accu-Check, Roche). Mice with fasting blood glucose > 14 mM was considered as diabetic.

At the end of the treatment (day 15), mice were weighed and anesthetized with sodium pentobarbital (60 mg/kg i.p.). Blood was rapidly collected by cardiac puncture in syringes containing EDTA. Blood samples were centrifuged (1 min, 8000 g), plasma collected, aliquoted, and snap frozen in liquid nitrogen. Plasma parameters were assayed using commercially available kits according to the manufacturers’ instructions: triglycerides (TG: Triglycerides PAP, bioMérieux, Marcy l'Etoile, France), cholesterol (Cholesterol RTU, bioMérieux), HDL cholesterol (HDL-Cholestérol direct, bioMérieux), and LDL cholesterol (LDL-C) level was determined using the formulae:[12]

$\text{LDL}-\text{C}=\text{TC}-\left(\text{HDL}-\text{C}+\frac{\text{TG}}{\text{n}}\right)$

n = 2 when values are expressed in mmol/L and n = 5 when values are expressed in g/L

LDL-C: LDL cholesterol; TC: total cholesterol; HDL-C: HDL cholesterol; TG: triglycerides

### Supernatant preparation

The tissue samples of liver were quickly removed, weighed, perfused immediately with ice-cold saline (0.85%, w/v NaCl), and homogenized in chilled phosphate buffer (0.1 M, pH 7.4) containing potassium chloride (1.17%, w/v). The homogenate was centrifuged (800 g, 5 min, 4 °C) to remove debris. The supernatant so obtained was centrifuged at 10,000 g for 20 min at 4 °C to get postmitochondrial supernatant preparation, which was used to assay the CAT, SOD, and GPx activities.

### Determination of the extract effect on lipid peroxidation in liver

Lipid peroxidation was estimated by thiobarbituric acid (TBA) reaction with malondialdehyde (MDA).[13] To 1 mL of supernatant, 0.5 mL of 30% trichloroacetic acid (TCA) was added followed by 0.5 mL of 0.8% TBA. The tubes were kept in a shaking water bath for 30 min at 80 °C. After 30 min of incubation the tubes were taken out and kept in ice-cold water for 10 min. These were then centrifuged at 800 g for 15 min. The absorbance of supernatant was read at 540 nm at room temperature against an appropriate blank. The concentration of MDA was measured from the standard calibration curve (prepared by) using tetraethoxypropane. Lipid peroxidation was expressed as nanomoles of MDA per milligram of protein.

### Determination of the extract effect on antioxidant enzyme activities in the liver

#### Superoxide dismutase activity

The SOD activity was measured according to the method used by Marklund and Marklund.[14] The enzyme activity was expressed as units/mg protein and one unit of enzyme is defined as the enzyme activity that inhibits autoxidation of pyrogallol by 50%.

#### Catalase activity

To estimate the CAT activity the reaction mixture consisted of 1.95 mL phosphate buffer (0.1 M, pH 7.4), 1.0 mL hydrogen peroxide (H_2_O_2_) (0.019 M), and 0.05 mL of supernatant in a final volume of 3 mL. Changes in absorbance were recorded at 240 nm. The enzyme activity was calculated as nanomoles of H_2_O_2_ consumed/min/mg protein. The protein content of the supernatant was determined using the method with copper sulphate.[15]

#### Glutathione peroxidase activity

To estimate the GPx activity, the reaction mixture consisted of 1.65 mL phosphate buffer (0.1 M, pH 7.4), 0.1 mL EDTA (0.5 mM), 0.05 mL oxidized glutathionee (1 mM), 0.1 mL NADPH (0.1 mM), and 0.1 mL supernatant in a total volume of 2 mL. The disappearance of NADPH at 340 nm was recorded at 25 °C. The enzyme activity was calculated as nanomol of NADPH oxidized/min/mg protein using molar extinction coefficient of 6.22 × 10^3^/M/cm.[16]

## Results

### Body weight, food, and water intake

**Figure 1:**
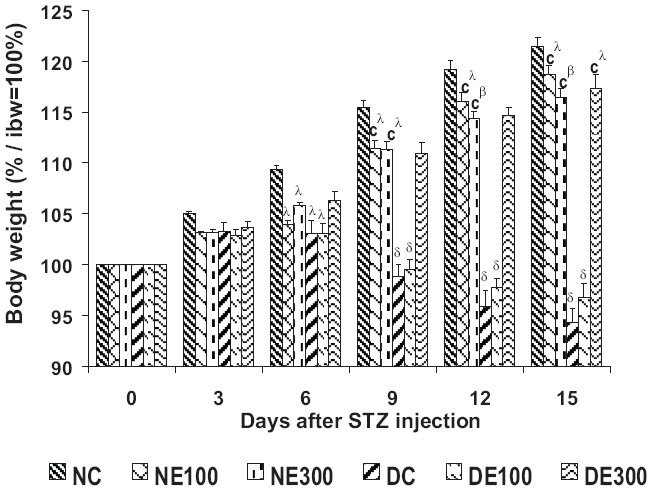
Body weight mass* over 15 days of treatment with *Terminalia glaucescens* leaf extract *Expressed as percentage of initial values ibw = 100% Data are mean ± SEM, (n = 8 per group). Significant difference: ^λ^*P* < 0.05, ^β^*P* < 0.01, ^δ^*P* < 0.001 compared with NC values; ^c^*P* < 0.001 compared with DC values

**Figure 2:**
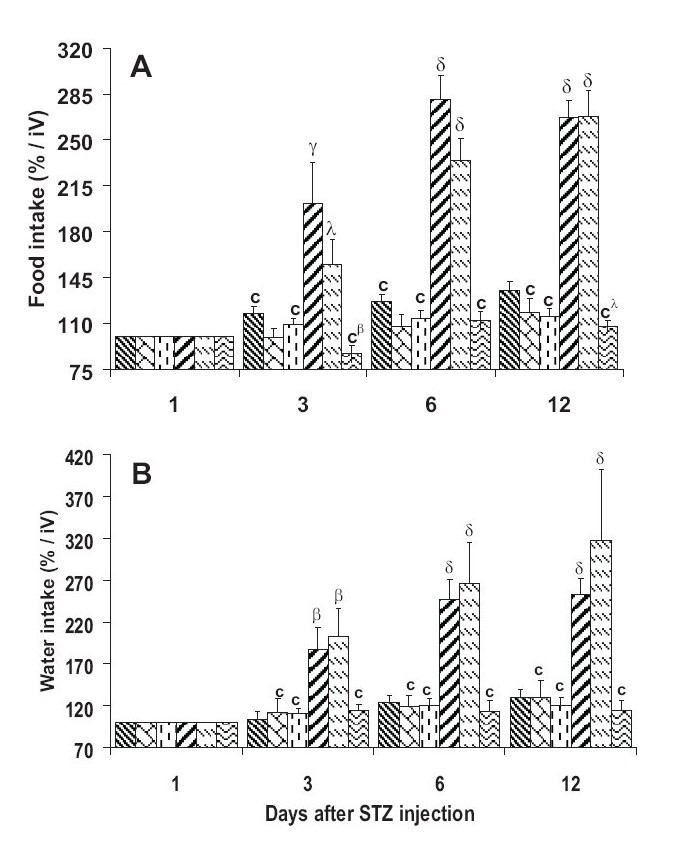
Food (A) and water (B) intake* over 12 days of treatment with *Terminalia glaucescens* leaf extract *Expressed as initial values iV = 100% Data are mean ± SEM, (n = 8 per group). Significant difference: ^λ^*P* < 0.05, ^β^*P* < 0.01, ^δ^*P* < 0.001 compared with NC values; ^c^*P* < 0.001 compared with DC values

Body weight of all groups was not significantly different from NC group before STZ injection. The body weight gain progressively decreased in DC and diabetic group treated with 100 mg/kg b.w. extract (DE100). The decrease was significant on day 9 and beyond: DC - 28% and DE100 - 25% (*P* < 0.001) on day 15, compared to NC [Figure 1]. NC, normal treated (NE100 and NE300), and 300 mg/kg extract pretreated diabetic (DE300) mice body weight gain increased progressively, but the body weight gain of extract-treated animals was markedly slowed down. Furthermore, DC and DE100 presented significant (*P* < 0.01) increase in food and water intake on day 3 which became more marked on day 12, whereas other groups (NC, NE100; NE300, and DE300) did not exhibit very significant variation [Figure 2A and B]. However, the extract slowed down food and water intake.

### Plasma metabolites

#### Blood glucose

**Figure 3:**
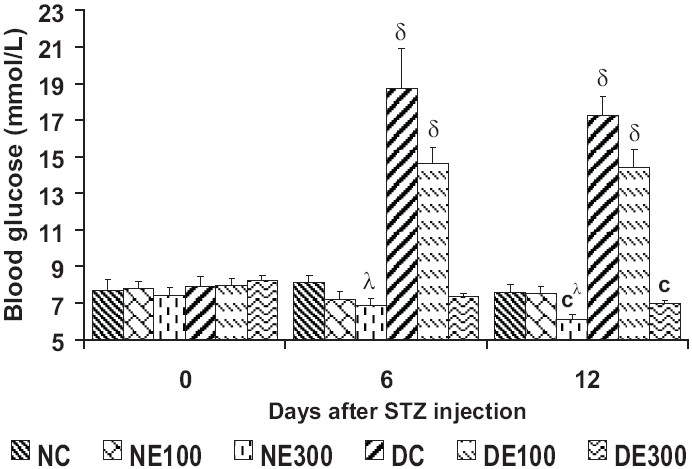
Blood glucose levels over 12 days of treatment with *Terminalia glaucescens leaf extract* Data are mean ± SEM, (n = 8 per group). Significant difference: ^λ^*P* < 0.05, ^δ^*P* < 0.01, ^c^*P* < 0.001 compared with DC values

In normal mice, *T. glaucescens* treatment resulted in the decrease of blood glucose levels in a dose dependent manner, 9% (*P* < 0.05) in NE300 [Figure 3]. Blood glucose level was very significantly (*P* < 0.001) increased on the day 6 and beyond in DC. The 300 mg/kg extract produced a significant fall compared to DC, so that glycemia was comparable to NC. Extract of 100 mg/kg b.w. did not show marked protective effect in mice against STZ-induced hyperglycemia.

#### Plasma lipids

In DC, blood levels of TG, cholesterol, and LDL -C significantly increased, 58%, 29%, and 18% (*P* < 0.05), respectively, whereas DC and DE100 HDL -C significantly decreased, −35% and −40% (*P* < 0.05), respectively [Table 1]. In normal mice these plasma parameters were not notably affected by the extract. In DE300 the extract prevented TG increase, reduced cholesterol and LDL-C increase, and prevented HDL-C decrease.

**Table 1:** Plasma lipids** after 15 days of treatment† with ***Terminalia glaucescens*** leaf extract

	*NC*	*NE100*	*NE300*	*DC*	*DE100*	*DE300*
TG (mmol/L)	0.889 ± 0.040	0.941 ± 0.060	0.871 ± 0.060	1.407 ± 0.185*	1.292 ± 0.162	1.022 ± 0.032*^a^
Cholesterol (mmol/L)	1.334 ± 0.111	1.320 ± 0.170	1.141 ± 0.066	1.724 ± 0.119*	1.515 ± 0.096	1.440 ± 0.138
HDL-c (mmol/L)	0.065 ± 0.004	0.068 ± 0.002	0.077 ± 0.007	0.042 ± 0.002*	0.039 ± 0.006*	0.063 ± 0.008
LDL-c (mmol/L)	0.860 ± 0.109	0.714 ± 0.197	0.682 ± 0.092	1.015 ± 0.141	0.816 ± 0.178	0.766 ± 0.102

Normal control (NC); normal mice treated with 100 mg/kg (NE100) or 300 mg/kg (NE300) extract diabetic control (DC); diabetic-test mice treated with 100 mg/kg (DE100) or 300 mg/kg (DE300) extract

** Plasma lipids: Triglycerides: TG, cholesterol, HDL cholesterol: HDL-C, LDL cholesterol: LDL-C;

† once daily, Data are mean ± SEM (n = 8 per group). Significant difference:

* *P* < 0.05 compared with NC values;

a *P* < 0.05 compared with DC values

### Lipid peroxidation in the liver

**Figure 4:**
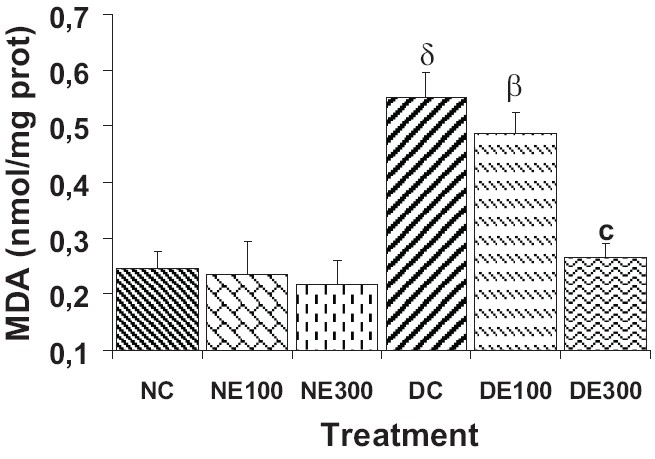
Lipid peroxidation* in mice liver after 15 days of treatment (once daily) with *Terminalia glaucescens* leaf extract *Expressed as malondialdehyde, MDA, concentration: nmol/mg of protein) Data are mean ± SEM, (n = 8 per group). Significant difference: ^β^*P* < 0.01, ^δ^*P* < 0.001 compared with NC values; ^c^*P* < 0.001 compared with DC values

The MDA level in DC and DE100 mice liver homogenates was very significantly higher than NC MDA, 129% (*P* < 0.001) and 104% (*P* < 0.01), respectively [Figure 4]. The 300 mg/kg extract prevented the increase so that the DE300 MDA level was similar to NC. The extract did not apparently affect lipid peroxidation in normal animals.

### Antioxidant enzyme activities in the liver

The activities of the three antioxidant enzymes –SOD, CAT, and GPx – in the liver homogenates markedly decreased in DC and DE100 mice, −49% and −42% (*P* < 0.01) for SOD, −44% and −43% (*P* < 0.05) for CAT, and −31% and −35% (*P* < 0.05) for GPx, respectively [Table 2].

**Table 2:** Antioxidant enzyme*** activities in the liver after 15 days of treatment† with Terminalia glaucescens leaf extract

	*NC*	*NE100*	*NE300*	*DC*	*DE100*	*DE300*
SOD (U/mg prot)	11.98 ± 0.96	12.69 ± 0.88	13.94± 1.20	6.31 ± 0.47*	7.12 ± 0.49**	10.94 ± 0.59^b^
CAT (nmol H_2_O_2_/min/mg prot)	280.08 ± 29.99	291.10 ± 27.42	351.03 ± 46.52	157.75 ± 9.53*	164.16 ± 6.76*	286.47 ± 16.87^a^
GPx (nmol NADPH/min/mg prot)	145.91 ± 11.38	151.78 ± 16.96	160.95 ± 20.20	100.66 ± 3.75*	94.87 ± 7.51*	131.11 ± 6.69^c^

*** Superoxide dismutase: SOD, catalase: CAT, glutathion peroxidase: GPx;

† once daily, Normal control (NC); normal mice treated with 100 mg/kg (NE100) or 300 mg/kg (NE300) extract; diabetic control (DC); diabetic-test mice treated with 100 mg/kg (DE100) or 300 mg/kg (DE300) extract, Data are mean ± SEM (n = 8 per group). Significant difference

* *P* < 0.05

** *P* < 0.01 compared with NC values

a *P* < 0.01

b *P* < 0.05

c *P* < 0.001, compared with DC values

In the DE300 liver homogenates, the extract prevented the loss of enzyme activities which were finally comparable to NC values. The plant extract enhanced antioxidant activities in the liver homogenates of the normal mice.

## Discussion

This study assessed the antioxidant properties of methanol–methylene chloride extract of *T. glaucescens* leaf on the liver of STZ-induced stressed mice. Multiple i.p. administration of STZ (45 mg/kg, once daily for five consecutive days) effectively induced diabetes in normal fasted mice as reflected by high glycemia, polyphagia, polydipsia, and body weight loss compared with NC mice. The hyperglycemia and diabetes were imputed to the selective destruction of pancreatic β-cells that secrete insulin.[17] Diabetes mellitus in rodents is a reliable and useful model for rapid observation of the protective effects of investigated agents on diabetes-induced damage.[7]

The STZ diabetic mice exhibited persistent hyperglycemia which is the main diabetogenic factor and contributes to the increase in oxygen free radicals by autoxidation of glucose.[18] Hyperglycemia also generates reactive oxygen species, which in turn, cause lipid peroxidation and membrane damage.[19] Diabetes increases oxidative stress in many organs, especially in the liver,[20] and thus may play a role in the pathogenesis and progression of diabetic tissue damage.[21] In this study, the level of MDA, an indicator of free radical generation and end product of lipid peroxidation, significantly increased in the untreated and 100 mg/kg extract treated diabetic mice liver. Lipid peroxidation is a commonly used index of increased oxidative stress and subsequent cytotoxicity. The increase in MDA level in diabetes mellitus suggests that hyperglycemia has induced peroxidative reaction in lipids.[22] With 300 mg/kg extract, the decrease of the MDA level in diabetic test mice suggests that *T. glaucescens* extract might protect against lipid peroxidation and diabetic oxidative stress.

In this study, the lipid peroxidation induced by STZ was associated with increased cholesterol, LDL cholesterol, triglyceridemia, decreased HDL-C, and key antioxidant enzymes (SOD, CAT, and GPx) in the DC and 100 mg/kg treated (DE100) diabetic mice. This result suggests that the extract at 100 mg/kg b.w. was not efficient enough to protect β-cell against the cytotoxic effect of STZ-mediated reactive oxygen species. Since β-cell necrosis and apoptosis is the core of the pathophysiology of diabetes mellitus, this might explain the diabetes mellitus state in these groups.

In 300 mg/kg extract treated diabetic mice (DE300) lipid peroxidation was associated with the decrease of LDL-C and TG, and the increase of HDL-C and key antioxidants. Lowering of LDL-C and TG levels with the enhancing of HDL-C level, is important for preventing high mortality lifestyle-related cardiovascular diseases. *T. glaucescens* can therefore be expected to help to prevent such diseases and this may explain the use of this plant in the treatment of diabetes and hypertension by tradipractitioners. The decrease in the activity of antioxidant enzymes could lead to an excess availability of the superoxide anion (O_2_^−^) and H_2_O_2_ in biological systems, which in turn, generate hydroxyl radicals resulting in initiation and propagation of lipid peroxidation. In diabetic mice, the extract (300 mg/kg b.w.) increased the activity of antioxidants and may help to control free radicals. SOD protects the cell against the toxic effect of superoxide anion radicals. The increased SOD activity accelerates dismutation of superoxide radicals to H_2_O_2_, which is removed by CAT.[10] GPx is an important antioxidant enzyme that plays a role in the elimination of H_2_O_2_ and lipid hydroperoxides and reduces peroxides by using reduced glutathione as a hydrogen donor.[23] The increase of SOD activity by the extract (300 mg/kg) might be attributed to the inhibition of active oxygen species generation from autoxidation of glucose generated by STZ action, while the increase of the CAT activity in the liver might indicate a high degree of oxidative stress resulting in the increase of endogenous H_2_O_2_. *T. glaucescens* extract contains chemical components such as tannins, alkaloids, flavonoids, and saponosides.[10] Flavonoids exhibit potent antioxidative and free radical scavenging activities.[24] Thus, the antioxidant activities of *T. glaucescens* extract are probably due to the presence of flavonoids or other compounds.

The present results indicate that the methanol–methylene chloride extract of *T. glaucescens* leaf at 300 mg/kg b.w. dose improved the SOD, CAT, and GPx activities, resulting in lower MDA level, and protected against STZ-induced oxidative stress in mice. The antihyperglycemic and antioxidant effects of *T. glaucescens* extract in STZ-induced diabetes could explain the traditional use of this plant for treatment or management of obesity and diabetes.
